# Deaggregation and Crystallization Inhibition by Small Amount of Polymer Addition for a Co-Amorphous Curcumin-Magnolol System

**DOI:** 10.3390/pharmaceutics13101725

**Published:** 2021-10-18

**Authors:** Jiawei Han, Luyuan Li, Meiling Su, Weili Heng, Yuanfeng Wei, Yuan Gao, Shuai Qian

**Affiliations:** School of Traditional Chinese Pharmacy, China Pharmaceutical University, Nanjing 211198, China; 3119020117@stu.cpu.edu.cn (J.H.); 3219020515@stu.cpu.edu.cn (L.L.); 3120020128@stu.cpu.edu.cn (M.S.); 1520200005@cpu.edu.cn (W.H.); weiyuanfengyuer@yeah.net (Y.W.)

**Keywords:** curcumin, magnolol, co-amorphous, deaggregation, dissolution, physical stability

## Abstract

Different from previously reported co-amorphous systems, a co-amorphous curcumin-magnolol (CUR-MAG CM) system, as compared with its crystalline counterparts, exhibited decreased dissolution due to its aggregation during dissolution. The main purpose of the present study is to deaggregate CUR-MAG CM to optimize drug dissolution and explore the deaggregation mechanism involved. Herein, a small amount of polymer (HPMC, HPC, and PVP K30) was co-formulated at 5% (*w*/*w*) with CUR-MAG CM as ternary co-amorphous systems. The polymer addition changed the surface properties of CUR-MAG CM including improved water wettability enhanced surface free energy, and hence exerted a deaggregating effect. As a result, the ternary co-amorphous systems showed faster and higher dissolution as compared with crystalline CUR/MAG and CUR-MAG CM. In addition, the nucleation and crystal growth of dissolved CUR and MAG molecules were significantly inhibited by the added polymer, maintaining a supersaturated concentration for a long time. Furthermore, polymer addition increased the T_g_ of CUR-MAG CM, potentially involving molecular interactions and inhibiting molecular mobility, resulting in enhanced physical stability under 25 °C/60% RH and 40 °C/75% RH conditions. Therefore, this study provides a promising strategy to optimize the dissolution and physical stability of co-amorphous systems by deaggregation and crystallization inhibition via adding small amounts of polymers.

## 1. Introduction

Most drug candidates under development are poorly soluble in water, which is related to various pharmaceutical performance problems [1,2]. Enhancing the solubility/dissolution of these drugs has become a vital issue for pharmaceutical enterprises to develop effective medicines with suitable dosage forms for patients [3]. Amorphization by disordering crystal lattice is a well-known approach to overcome the solubility/dissolution defects of poorly soluble drugs [4,5,6]. However, the application of amorphous systems is severely limited due to the inherent thermodynamic instability and devitrification risk during processing, storage, and dissolution [7,8,9], resulting in the loss of amorphous advantages. Therefore, it is crucial to seek effective approaches to stabilize amorphous drugs.

In recent years, co-amorphous drug delivery systems have aroused wide attention for stabilizing neat amorphous drugs and providing solubility/dissolution advantages (such as the faster dissolution rate and enhanced supersaturation ability) over corresponding crystalline and amorphous drugs [10]. A co-amorphous system is prepared by amorphizing a drug and a small molecule excipient (e.g., glibenclamide-arginine [11] and griseofulvin-tryptophan [12]) or a drug and another drug (e.g., docetaxel-bicalutamide [13] and ezetimibe-simvastatin [14]), to form a single amorphous phase in a certain stoichiometric ratio. In addition, a co-amorphous drug delivery system has become an effective alternative strategy to overcome the limitations of amorphous solid dispersion (ASD). Different from polymer-based ASD, a co-amorphous system possesses lower volume/mass of dosage with superior physical stability [6,15]. Furthermore, drug combinations are also intended to form co-amorphous systems in order to enhance solubility/dissolution of poorly soluble drugs, and also achieve potential combination therapy [16], such as ketoprofen-ethenzamide [17] and famotidine and ibuprofen [18].

**Figure 1:**
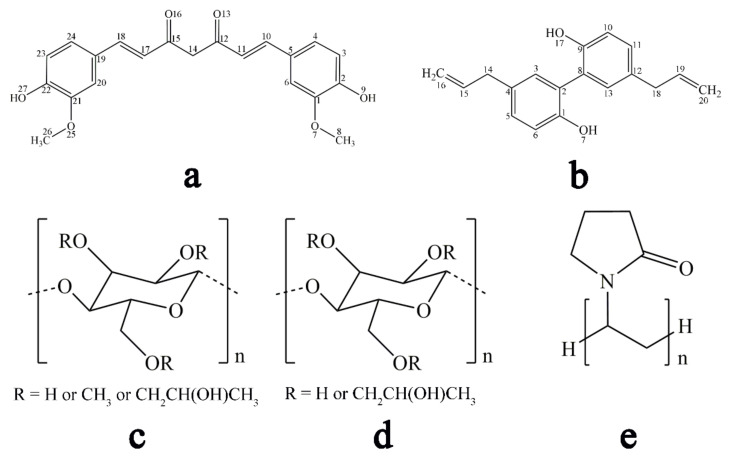
Chemical structures of the model drugs: (**a**) CUR; (**b**) MAG and commonly used polymeric stabilizers; (**c**) HPMC; (**d**) HPC; and (**e**) PVP K30.

In the present study, co-amorphous curcumin-magnolol was designed based on their solubility/dissolution defects and potential drug combination advantages. Curcumin (CUR, Figure 1a) and magnolol (MAG, Figure 1b) have gained increasing interest because of their multiple biological activities (such as antioxidant, anticancer, antimicrobial, and anti-inflammatory) [19,20,21,22,23]. Moreover, the two drugs have generated combined therapeutic effects in cardiovascular diseases, Alzheimer’s disease, and neurodegenerative diseases [24,25,26]. However, CUR shows poor oral absorption mainly because of low aqueous solubility/dissolution (0.96 µg/mL in simulated gastric fluid) [8]. In addition, it is prone to phase II metabolism by β-glucuronidase in the gastrointestinal tract [27], while MAG also belongs to poorly soluble drugs and has been proven to be an inhibitor of β-glucuronidation with high inhibitory activity [28,29]. Thus, such a co-amorphous system was designed and prepared.

Whereas, unlike high solubility/dissolution and physical stability of co-amorphous systems previously reported, co-amorphous CUR-MAG (CUR-MAG CM) unexpectedly exhibited much lower dissolution as compared with their crystalline counterparts due to its aggregation during dissolution. Since polymeric excipients have potential to enhance the solubility/dissolution of poorly soluble drugs and stabilize amorphous drugs, the question is whether co-formulating with only a small amount of polymer could deaggregate CUR-MAG CM to optimize the dissolution, and improve its physical stability? The aim of this study was to answer this question and explore the internal mechanism for deaggregation effect using small amounts of polymers.

## 2. Materials and Methods

### 2.1. Materials

Crystalline CUR (>98%) and crystalline MAG (>98%) were acquired from Aladdin Biochemical Technology Co., Ltd. (Shanghai, China) and Tianben Biological Engineering Co., Ltd. (Xi’an, China), respectively. Hydroxypropyl methyl cellulose (HPMC), hydroxypropyl cellulose (HPC), polyvinyl pyrrolidone (PVP) K30, microcrystalline cellulose (MCC), kollidon VA64 (VA64), and plasdone S630 (S630) were purchased from Colorcon Co., Ltd. (Philadelphia, PA, USA). The chemical structures of CUR, MAG, HPMC, HPC, and PVP K30 are given in Figure 1. Tween-80 and phosphoric acid were supplied from Nanjing Chemical Reagent Co., Ltd. (Nanjing, China). Methanol and acetonitrile of chromatographic grade were purchased by Anpel Scientific Instrument Co., Ltd. (Shanghai, China). Deionized water was prepared by an ultrapure water purification system (OLABO, Boke Instruments, Jinan, China).

### 2.2. Drug-Drug/Polymer Miscibility Using Solubility Parameters (δ)

The solubility parameter (*δ*) is calculated via Fedors method or Hoftyzer–Van Krevelen method [30]. Among them, Hildebrand solubility parameter (Equation (1)) is calculated via the Fedors method. Where cohesive energy density (CED), *V_m_*, and *ΔE_v_* represent the cohesive energy per unit volume, the molar volume, and the energy of vaporization, respectively [30,31]. In addition, Hansen solubility parameter (Equation (2)) uses the existing theories of Van Kravlen and Hoftyzer. Where *δ_t_* is the total solubility parameter, *δ_d_* is the dispersive solubility parameters, *δ_p_* is the polar solubility parameters, and *δ_h_* is the hydrogen bonding solubility parameters. Then, these three parameters can be obtained via Equation (3). In these equations, *F_di_*, *F_pi_*, and *E_hi_* represent the group contributions of various substances. In addition, V refers to the group contribution to molar volume [32]. When the solubility parameter difference (*Δδ*) of two components is less than 7.0 MPa^1/2^, there is considered to be good miscibility between them [30,33] as follows:

(1) $$\delta ={\left(CED\right)}^{0.5}={\left(\frac{\Delta E_{V}}{V_{m}}\right)}^{0.5}$$

(2) $$\delta_{t}^{2}=\delta_{d}^{2}+\delta_{p}^{2}+\delta_{h}^{2}$$

(3) $$\delta_{d}=\frac{\sum F_{di}}{V},\delta_{p}=\frac{\sqrt{\sum F_{pi}^{2}}}{V},\;\;\delta_{h}=\sqrt{\frac{\sum E_{hi}}{V}}$$

### 2.3. Preparation of Co-Amorphous Systems

A thermogravimetric analyzer (Q500, TA Instruments, New Castle, DE, USA) was used to investigate degradation temperatures of crystalline CUR and crystalline MAG in order to ensure no thermal degradation during preparation of co-amorphous CUR-MAG (CUR-MAG CM). Crystalline CUR and crystalline MAG (about 3 mg) were accurately weighed in aluminum pans and heated from 30 to 250 °C at a rate of 10 °C/min under a constant velocity of nitrogen (40 mL/min).

Then, CUR-MAG CM was prepared by quench cooling. In brief, a total of 1000 mg of CUR and MAG (molar ratio of 1:1, 580.4 mg of CUR, 419.6 mg of MAG) was evenly mixed on the oscillator for 10 min. The homogeneous physical mixture (CUR-MAG PM) was co-melted in an oil bath at 130 °C for 5 min and rapidly cooled using liquid nitrogen, forming the co-amorphous system. On the basis of their solubility parameters, six polymers (i.e., HPMC, HPC, PVP K30, MCC, VA64, and S630) were selected and co-formulated with CUR-MAG CM as ternary co-amorphous systems due to the good miscibility between CUR/MAG and the selected polymers. The ternary co-amorphous systems (i.e., CUR-MAG-HPMC CM, CUR-MAG-HPC CM, and CUR-MAG-PVP K30 CM) were also prepared by melting the above CUR-MAG PM with a small amount of polymer excipient (5% of HPMC, HPC, or PVP K30, w_polymer_/w_drug_) and quenching with liquid nitrogen. Meanwhile, other ternary co-amorphous systems were also prepared with MCC, VA64, and S630 in the same way (i.e., CUR-MAG-MCC CM, CUR-MAG-VA64 CM, and CUR-MAG-S630 CM). The obtained samples were ground with an agate mortar, sieved through 80 meshes, and stored in a desiccator above silica gel and anhydrous calcium chloride before analysis.

### 2.4. X-ray Powder Diffraction (XRPD)

XRPD diffractograms of the samples were acquired using an X-ray diffractometer (D/max 2500, Rigaku Co., Tokyo, Japan) with Cu-Kα radiation of 1.5406 Å. The diffractometer was operated with a fixed tube current (100 mA) and a voltage (40 kV). A graphite monochromator was employed to monochromate the X-ray beam, and a standard scintillation counter simultaneously served as the detector. The sample was scanned at a step size of 0.02° in the reflectance mode range of 5~40° (2*θ*).

### 2.5. Differential Scanning Calorimetry (DSC)

The samples were performed on a thermal analyzer system (DSC 250, TA Instruments, New Castle, DE, USA) for thermal analysis. Approximately 3 mg of the sample powders were sealed in an aluminum pan and heated to 250 °C at 10 °C/min under nitrogen flow with a rate of 50 mL/min. Data including the glass transition temperature (T_g_), recrystallization temperature (T_rc_), and melting temperature (*T_m_*) were analyzed using TRIOS software (version 5.0.0.44608). To obtain *T_g_s* of amorphous CUR and amorphous MAG, the thermal analyzer was used to prepare amorphous CUR and amorphous MAG, and to simultaneously determine their *T_g_s*. Crystalline CUR or crystalline MAG (approximately 3 mg) was sealed in an aluminum plate, melted at 5 °C above the melting point, and kept isothermal for 3 min in the DSC analyzer. Then, the sample plate was cooled to −25 °C and maintained for 3 min, and the sample was heated to 250 °C at a rate of 10 °C/min. *T_g_s* of amorphous CUR and amorphous MAG were analyzed using TRIOS software.

The *T_g_* of the co-amorphous system assuming free volume additivity can be predicted using the Fox equation (Equation (4)) [15,34] as follows:

(4) $$\frac{1}{T_{g12}}=\frac{w_{1}}{T_{g1}}+\frac{w_{2}}{T_{g2}}$$

where *T_g_*_12_ is the theoretical *T_g_* of the co-amorphous system, *T_g_*_1_ and *T_g_*_2_ are the *T_g_* of each component, *w*_1_ and *w*_2_ are their weight fractions. A positive or negative deviation from the theoretically calculated *T_g_* value reflects strong or weak intermolecular interactions between the components. Furthermore, a theoretical *T_g_* could also be calculated for q ternary co-amorphous systems using the Fox equation [10]. For example, in CUR-MAG-HPMC CM, the CUR-MAG CM was assumed as the first single “component”, and the added HPMC was treated as the second “component”, as has been previously reported [10,35].

### 2.6. Raman Spectroscopy

Raman spectra of the samples were monitored by an Invia Raman spectrometer (Renishaw Co., Ltd., Gloucestershire, UK) at a 785 nm excitation laser, and were scanned in a measurement range of 800~1800 cm^−1^ at a resolution of 4 cm^−1^. OMNIC software was used to analyze the measured spectral data.

### 2.7. Fourier Transform Infrared Spectroscopy (FTIR)

FTIR spectra of the samples were recorded on a FTIR spectrometer (IRAffinity-1S, Shimadzu Co., Ltd., Tokyo, Japan) equipped with LabSolutions IR software. Each sample (2 mg) was mixed with KBr (100 mg) for tablet compaction, and the tablet was scanned 45 times in the range of 400~4000 cm^−1^ at a resolution of 4 cm^−1^.

### 2.8. Solid-State ^13^C Nuclear Magnetic Resonance Spectroscopy (ss ^13^C NMR)

The ss ^13^C NMR spectra of the samples were determined using a Bruker AVANCE III 400 MHz wide-bore spectrometer (Bruker Analytik GmbH, Rheinstetten, Germany) equipped with a double-resonance CP-MAS probe head. The sample was placed in 4 mm ZrO_2_ rotors sealed with Kel-F1 to record the ^13^C NMR spectra with a sweep width of 50 kHz. The ss ^13^C NMR spectra were obtained using the magic-angle spinning (MAS frequency 14 kHz) technique and samples were collected 480 times. All spectra were referenced to an external sample of a-glycine at 176.03 ppm.

### 2.9. Dissolution under Supersaturated Conditions

Dissolution tests of crystalline CUR, crystalline MAG, CUR-MAG PM, CUR-MAG CM, CUR-MAG-HPMC CM, CUR-MAG-HPC CM, CUR-MAG-PVP K30 CM, CUR-MAG-MCC CM, CUR-MAG-VA64 CM, and CUR-MAG-S630 CM (equivalent to 400 mg of CUR or 289.2 mg of MAG, molar ratio of 1:1) were conducted in triplicates by a small-volume dissolution apparatus (RC806D, Tianda Tianfa Technology Co., Ltd., Tianjin, China) with a constant stirring of 100 rpm at 37 °C. The dissolution tests were carried out in 200 mL 0.1 M HCl buffer (pH 1.2) and 0.2 M phosphate buffer (pH 6.8) with 0.5% Tween-80. At predetermined time points (10, 20, 30, 45, 60, 90, 120, 240, 360, and 480 min), 2 mL aliquots were collected and an equal volume of fresh medium was immediately added to the medium. After filtration using a 0.45 μm membrane and dilution with an equal volume of methanol, the concentration of CUR and MAG was analyzed at 35 °C by the HPLC system (LC-2010A, Shimadzu Co., Ltd., Tokyo, Japan) with a Kromasil^®^ C18 column (5 μm, 250 × 4.6 mm). The mobile phase (60:40, acetonitrile to 0.3% phosphoric acid solution) was pumped up at the speed of 1 mL/min for 18 min. The wavelengths for detecting CUR and MAG were set at 430 and 294 nm, respectively.

### 2.10. Contact Angle Measurements and Surface Free Energy Calculation

The contact angle measurements of the co-amorphous samples were conducted by sessile drop method using a contact angle apparatus (OCA15EC, Dataphysics Ltd., Filderstadt, Germany) with different test liquids (i.e., water, glycerol, and diiodomethane) [36,37]. The contact angles for both sides of each drop were measured between the liquid and tablet surface. Furthermore, the contact angle measurement is the most common method to calculate surface free energy.

The surface free energy parameters were calculated from the contact angle data of the test liquids, which were analyzed by van Oss’s acid-base method. According to van Oss and co-workers to interfacial tensions, the solid surface free energy is composed of nonpolar Lifshitz-van der Waals ($\gamma_{SV}^{LW}$) and polar acid-base ($\gamma_{SV}^{AB}$) interactions. The $\gamma_{SV}^{AB}$ interaction is attributed to the electron-donor ($\gamma_{SV}^{-}$) and the electron-acceptor ($\gamma_{SV}^{+}$) interactions. Thus, the total surface free energy ($\gamma_{SV}^{tot}$) of the solid can be expressed as Equation (5) [36,38,39] as follows:

(5) $$\gamma_{SV}^{tot}=\gamma_{SV}^{LW}+\gamma_{SV}^{AB}=\gamma_{SV}^{LW}+2\sqrt{\gamma_{SV}^{+}\;\gamma_{SV}^{-}}$$

The relationship between the solid-liquid interfacial tension ($\gamma_{SL}$) as a function of the components and parameters of the surface tension of the solid and liquid can be expressed as Equation (6) [38,40]:

(6) $$\gamma_{SL}=\gamma_{SV}+\gamma_{LV}-2\sqrt{\gamma_{LV}^{LW}\;\gamma_{SV}^{LW}}-2\sqrt{\gamma_{LV}^{+}\;\gamma_{SV}^{-}}-2\sqrt{\gamma_{LV}^{-}\;\gamma_{SV}^{+}}$$

where $\gamma^{LW}$ is the Lifshitz-van der Waals component of the surface free energy of solids or liquids, $\gamma^{-}$ and $\gamma^{+}$ are the electron-donor and electron-acceptor parameters of the acid-base components of the solid (*SV*) or liquid (*LV*) surface free energy, respectively.

Combining with the Young equation (Equation (7)):

(7) $$\gamma_{LV}\cos\theta =\gamma_{SV}-\gamma_{SL}$$

and the work of the liquid adhesion to solid surface (Adhesion work, *W_A_*) equation (Equation (8)):

(8) $$W_{A}=\gamma_{LV}\left(\cos\theta +1\right)$$

The *W_A_* can be further expressed by Equation (9) [36,38,39,40]:

(9) $$W_{A}=\gamma_{LV}\left(\cos\theta +1\right)=2\sqrt{\gamma_{LV}^{LW}\;\gamma_{SV}^{LW}}+2\sqrt{\gamma_{LV}^{+}\;\gamma_{SV}^{-}}+2\sqrt{\gamma_{LV}^{-}\;\gamma_{SV}^{+}}$$

### 2.11. Nucleation Inhibition of CUR and MAG by Polymer

Nucleation induction time (t_n_) was usually used to assess the effect of polymeric excipients on nucleation and crystallization of drug solutions [41]. In this study, supersaturated solutions of CUR-MAG CM, CUR-MAG-HPMC CM, CUR-MAG-HPC CM, and CUR-MAG-PVP K30 CM were prepared by adding 1 mL DMSO solution (i.e., dissolving the samples in DMSO solvent at the same CUR concentration equal to 2 mg/mL CUR) into 99 mL water with a constant stirring of 200 rpm at 25 °C. The turbidity-time profiles of the supersaturated solutions were determined in triplicate using a UV spectrophotometer (TU-1901, PGeneral Ltd., Beijing, China) at a wavelength of 600 nm (no UV absorption for both drugs and polymers).

### 2.12. Broadband Dielectric Spectroscopy (BDS)

The dielectric permittivity (ε*(ω) = ε′(ω) − iε″(ω)) measurements were conducted using the dielectric spectrometer (Novocontrol concept 40, Novocontrol Technologies GmbH & Co. KG, Montabaur, Germany). The spectrometer operated at the frequency range from 10^−2^ to 10^6^ Hz, and a Quatro Cryosystem equipped with a nitrogen gas cryostat controlled the temperature stability (better than 0.1 °C). The samples were placed between two stainless-steel flat electrodes of the capacitor with a 0.1 mm gap and mounted in a cryostat. Dielectric measurements on CUR-MAG CM, CUR-MAG-HPMC CM, CUR-MAG-HPC CM, and CUR-MAG-PVP K30 CM (5% of HPMC, HPC, or PVP K30 to drugs in the ternary co-amorphous systems, w_polymer_/w_drug_), were performed from 16 to 40 °C under dry nitrogen gas flow.

### 2.13. Physical Stability Evaluation

CUR-MAG CM, CUR-MAG-HPMC CM, CUR-MAG-HPC CM, and CUR-MAG-PVP K30 CM were stored under long-term condition (25 °C, 60% RH) and accelerated condition (40 °C, 75% RH) in a stability chamber (YSEI Experimental Instrument Co., Ltd., Chongqing, China) for 3 months. The samples were evaluated by XRPD on 7, 14, 21, 30, 60, and 90 days. The appearance of recrystallization peaks from the amorphous halo in the XRPD patterns reflected recrystallization.

## 3. Results and Discussion

### 3.1. Drug-Drug/Polymer Miscibility Using Solubility Parameters (δ)

Compounds are considered to be miscible when they have similar *δ* values, since energy requirements for mixing are compensated by the energy released in the interactions between the components (exothermic energy of mixing) [30]. For the theoretical miscibility assessment, if the solubility parameter difference (*Δδ*) is less than 7.0 MPa^1/2^, the components are considered to have good miscibility. If *Δδ* is less than 2.0 MPa^1/2^, the components may possess excellent miscibility and easily form a solid solution, while *Δδ* greater than 10 MPa^1/2^ indicates immiscibility between them [30,42]. Theoretical predicted *δ* using the Fedors and Hoftyzer–Van Krevelen methods are given in Supplementary Materials Table S1. The *Δδ* of 0.41 MPa^1/2^ between CUR and MAG suggested their excellent miscibility, and the *Δδ* values between CUR/MAG and each polymer were all less than 7.0 MPa^1/2^, indicating good miscibility between CUR/MAG and the selected polymers.

### 3.2. Preparation of Co-Amorphous Systems

The purpose of this section was to conduct safe preparation (without thermal decomposition) of CUR-MAG CM during melting and quench cooling. A thermogravimetric analysis (TGA) was used to investigate whether crystalline CUR and crystalline MAG underwent thermal degradation during the preparation process. As depicted in Figure S1, crystalline CUR showed no weight loss in the range from 30 to 198.1 °C, and crystalline MAG showed a steep descent in the TGA profile until 162.6 °C. The ranges of thermodynamic degradation were much higher than their melting points, as described in Section 3.3.2 (*T_m_* = 181.9 °C for CUR and *T_m_* = 101.4 °C for MAG, respectively). The results suggested that CUR-MAG CM could be safely prepared by quench cooling. Due to the good miscibility between CUR and MAG or between CUR/MAG and the polymers, the components easily formed a clarified solid solution. A polarizing light microscopy (PLM) observation demonstrated the complete amorphization of CUR-MAG CM and its ternary co-amorphous systems (Figure S2). At last, a higher yield rate of 98.15 ± 0.74% was obtained in CUR-MAG CM, and the content of CUR and MAG determined by the HPLC method was 0.573 ± 0.004 g/g for CUR and 0.408 ± 0.003 g/g for MAG in the final product of CUR-MAG CM (*n* = 3). In addition, all ternary co-amorphous systems also maintained high yield rates (Table S2).

### 3.3. Solid-State Characterization

#### 3.3.1. XRPD

**Figure 2:**
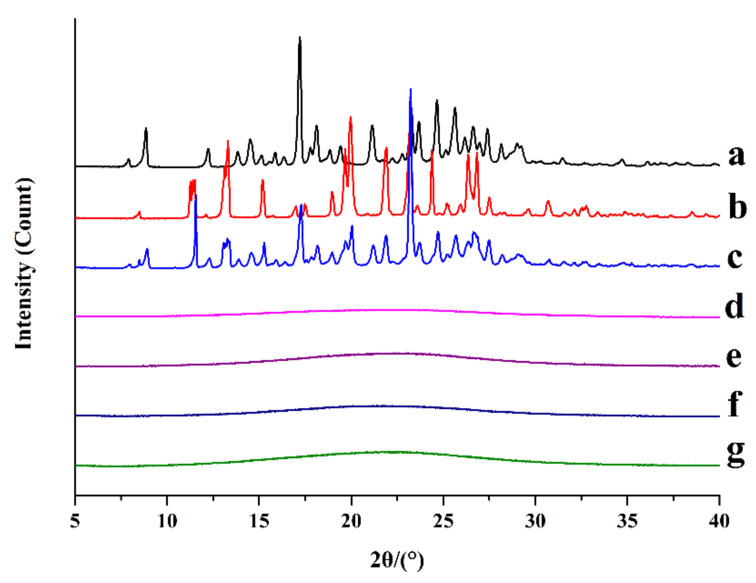
XRPD diffractograms: (**a**) crystalline CUR; (**b**) crystalline MAG; (**c**) CUR-MAG PM; (**d**) CUR-MAG CM; (**e**) CUR-MAG-HPMC CM; (**f**) CUR-MAG-HPC CM; (**g**) CUR-MAG-PVP K30 CM.

XRPD is used as a gold standard approach to characterize amorphous materials. The absence of crystallinity can be confirmed by spotting an amorphous halo pattern. As observed in Figure 2, crystalline CUR showed characteristically intense diffraction peaks at 7.90°, 8.91°, 12.26°, 13.82°, 14.52°, 17.16°, 18.18°, 19.43°, 21.15°, 23.30°, 23.69°, 24.71°, 25.63°, 26.65°, and 27.41° for 2*θ* scan (Figure 2a), which was consistent with the previous reports, indicating that crystalline CUR was the commercially used and most studied polymorph (form I) [43,44,45]. Correspondingly, crystalline MAG exhibited multiple distinct diffraction peaks ranging from 8 to 34 °C (Figure 2b). The diffractograms of crystalline CUR and MAG were compared with their standard patterns from the Cambridge Crystallographic Data Centre, and their XRPD diffractograms were consistent with their standard patterns (Figures S3 and S4). CUR-MAG PM still maintained the original characteristic peaks of the two crystalline components (Figure 2c). In contrast, for CUR-MAG CM, CUR-MAG-HPMC CM, CUR-MAG-HPC CM, and CUR-MAG-PVP K30 CM, the XRPD patterns exhibited the typical amorphous halo (i.e., absence of crystalline peaks), confirming complete amorphization (Figure 2d–g). The underlying mechanism of formation (ideal mixing or intermolecular interactions) was further verified by thermodynamic and spectral analysis.

#### 3.3.2. DSC

DSC measurements were performed to determine the thermodynamic properties of the prepared co-amorphous samples. The single T_g_ confirmed the formation of a homogeneous single-phase co-amorphous system. To reveal the relationship of potential interactions between the components in co-amorphous formulations, the experimental T_g_ was compared to the theoretically predicted T_g_ calculated from the Fox equation.

**Figure 3:**
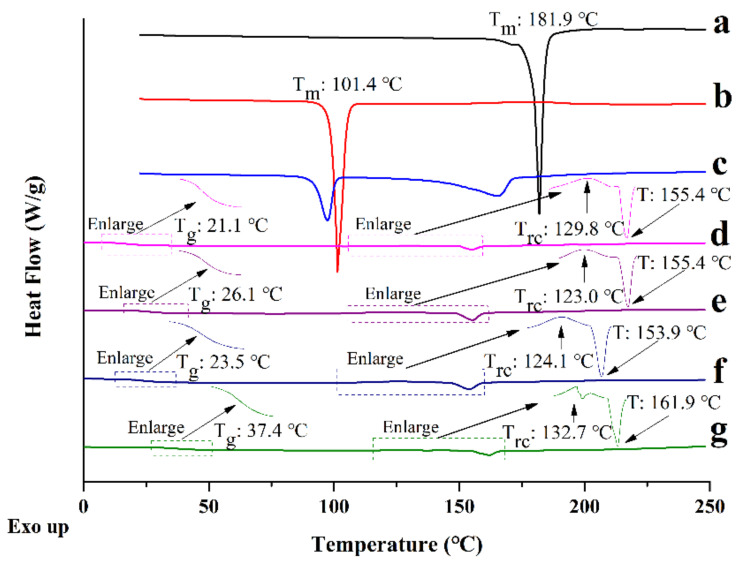
DSC thermograms: (**a**) crystalline CUR; (**b**) crystalline MAG; (**c**) CUR-MAG PM; (**d**) CUR-MAG CM; (**e**) CUR-MAG-HPMC CM; (**f**) CUR-MAG-HPC CM; (**g**) CUR-MAG-PVP K30 CM.

Crystalline CUR and crystalline MAG showed sharp endothermic peaks at 181.9 and 101.4 °C, which were their melting points (Figure 3a,b). For CUR-MAG PM, endothermic peaks were observed at 97.5 and 165.8 °C below their respective melting points (Figure 3c), which might be attributed to the low “eutectic-like” endothermic events [46]. However, CUR-MAG CM showed a single T_g_ at 21.1 °C, followed by recrystallizing at 129.8 °C, and remelting at 155.4 °C (Figure 3d). The ternary co-amorphous systems showed similar thermodynamic behavior as compared with CUR-MAG CM, except for exhibiting higher T_g_s (i.e., 26.1, 23.5, and 37.4 °C for CUR-MAG-HPMC CM, CUR-MAG-HPC CM, and CUR-MAG-PVP K30 CM, respectively) (Figure 3e–g). In general, the higher the T_g_ of the co-amorphous system, the higher the physical stability [1], implying potentially higher physical stability of the ternary co-amorphous systems than the binary CUR-MAG CM.

Amorphous CUR and amorphous MAG prepared in situ using DSC exhibited T_g_s of 74.6 and −16.4 °C, respectively (Figure S5). As shown in Figure 3 and Table 1, the experimental T_g_ of CUR-MAG CM was 21.1 °C, which was lower than the theoretically calculated T_g_ of 29.5 °C. The CUR-MAG CM revealed an 8.4 °C negative deviation of T_g_ from the theoretical value, indicating that there might be weak intermolecular interactions in CUR-MAG CM, which were further investigated by spectral analysis. The T_g_ values of HPMC, HPC, and PVP K30 were determined to be 140.4 [10], 86.2 [47] and 176.0 °C [48], as previously reported, which were used to calculate the theoretical T_g_s of the ternary co-amorphous systems. When CUR-MAG CM was regarded as a single amorphous “component”, CUR-MAG-HPMC CM, CUR-MAG-HPC CM, and CUR-MAG-PVP K30 CM showed theoretical T_g_ values of about 22.0, 21.9, and 22.0 °C, and their experimental T_g_ values exhibited 4.1, 1.6, and 15.4 °C positive deviation from the theoretical values, respectively (Figure 3 and Table 1). The positive deviation of T_g_ values indicated possible molecular interactions between CUR-MAG CM and the added polymers in ternary co-amorphous systems.

#### 3.3.3. Raman Spectroscopy

**Figure 4:**
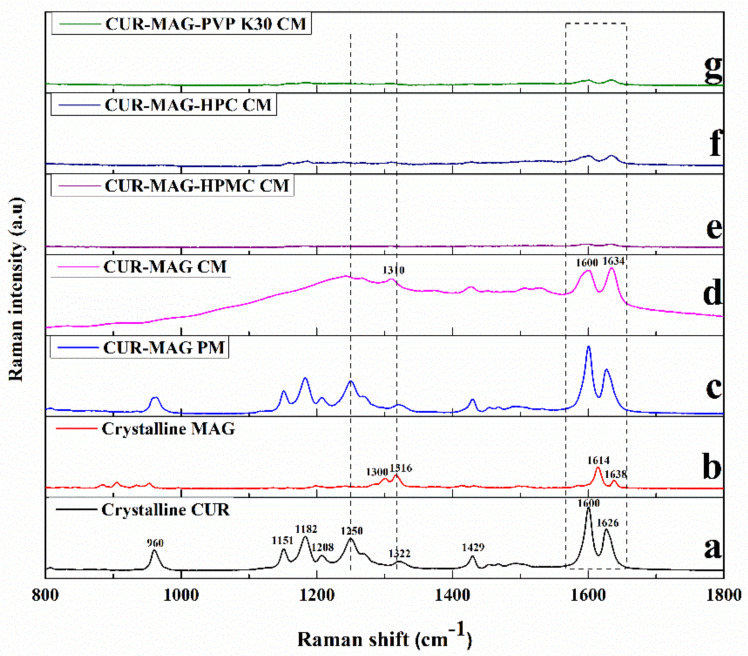
Raman spectroscopy: (**a**) crystalline CUR; (**b**) crystalline MAG; (**c**) CUR-MAG PM; (**d**) CUR-MAG CM; (**e**) CUR-MAG-HPMC CM; (**f**) CUR-MAG-HPC CM; (**g**) CUR-MAG-PVP K30 CM.

Raman spectroscopy was applied to analyze the molecular interactions between components. Crystalline CUR showed two characteristic doublet peaks at 1626 and 1600 cm^−1^, which were attributed to the vibrations of C=C and C=O groups, respectively [8,46,49,50]. It also exhibited a distinct characteristic peak at 1250 cm^−1^ due to the vibration of the phenolic OH group (Figure 4a) [8,46]. The Raman spectrum of crystalline MAG exhibited distinct absorption peaks at 1614 and 1316 cm^−1^ for the C=C and C-H vibrations, respectively (Figure 4b) [51]. For CUR-MAG PM, it performed the original absorption peaks of crystalline CUR and crystalline MAG (Figure 4c). However, in CUR-MAG CM, the vibration of the phenolic OH group (1250 cm^−1^) in CUR disappeared, the C=O vibration in CUR weakened and broadened at 1600 cm^−1^, and the absorption peak of the C-H (1316 cm^−1^) in MAG shifted to 1310 cm^−1^, implying potential interactions, such as hydrogen bond interactions occurring between CUR and MAG (Figure 4d). In addition, the ternary co-amorphous systems showed similar but significantly diminished spectra (especially at 1600 and 1634 cm^−1^) as compared with CUR-MAG CM, indicating possible interactions between CUR-MAG CM and the added polymer (Figure 4e–g).

#### 3.3.4. FTIR

**Figure 5:**
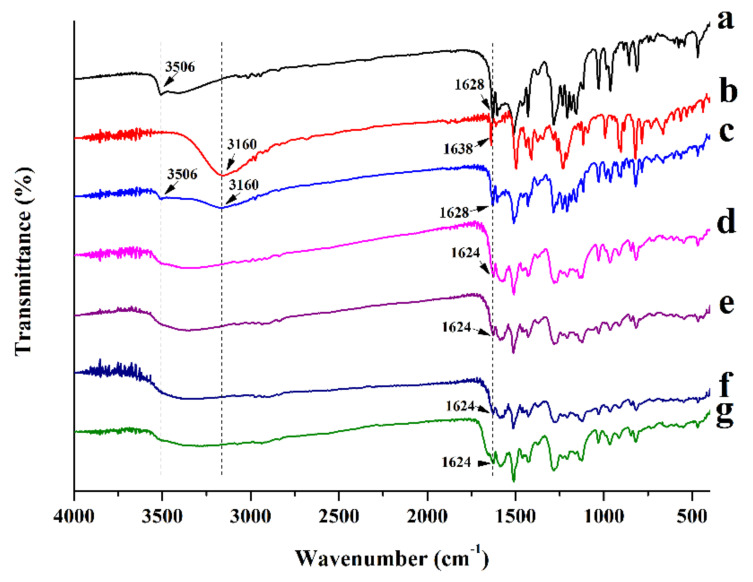
FTIR spectra: (**a**) crystalline CUR; (**b**) crystalline MAG; (**c**) CUR-MAG PM; (**d**) CUR-MAG CM; (**e**) CUR-MAG-HPMC CM; (**f**) CUR-MAG-HPC CM; (**g**) CUR-MAG-PVP K30 CM.

FTIR as a complementary technique for Raman spectroscopy, was conducted to further analyze potential intermolecular interactions between CUR and MAG in CUR-MAG CM or between the added polymer and the CUR-MAG CM system. As shown in Figure 5, crystalline CUR exhibited distinct characteristic peaks at 3506 cm^−1^ and 1628 cm^−1^ in its FTIR spectrum, which was attributed to the phenolic OH group and C=O group, respectively (Figure 5a). The FTIR spectrum of crystalline MAG showed distinct absorption bands at 3160 and 1638 cm^−1^ for the stretching of CH_2_ and C=C vibrations, respectively (Figure 5b). CUR-MAG PM showed the original absorption peaks from crystalline CUR and crystalline MAG (Figure 5c). However, in CUR-MAG CM, the absorption peaks of the phenolic OH group (3506 cm^−1^) disappeared and the C=O vibration in CUR slightly weakened and shifted from 1628 to 1624 cm^−1^, and the CH_2_ vibration (3160 cm^−1^) in MAG also disappeared, indicating an intermolecular hydrogen bond interaction between the two components (Figure 5d). In addition, similar spectra without obvious change could be observed between the ternary co-amorphous systems and the polymer-free CUR-MAG CM (Figure 5e–g), which suggested that hydrogen bond formation between CUR and MAG was maintained in a similar manner with or without polymer addition. On the basis of the Raman and FTIR results, the added polymer might involve molecular interactions in the ternary co-amorphous systems, but such interactions did not disturb the expected hydrogen bond formation between CUR and MAG [10].

#### 3.3.5. ss ^13^C NMR

**Figure 6:**
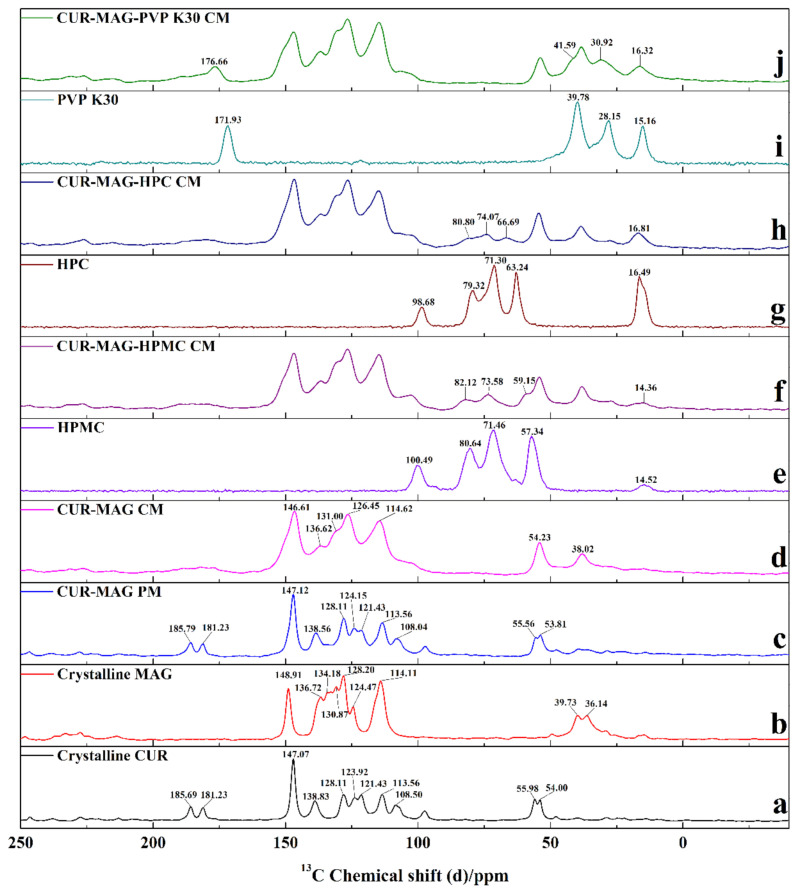
ss ^13^C NMR spectra: (**a**) crystalline CUR; (**b**) crystalline MAG; (**c**) CUR-MAG PM; (**d**) CUR-MAG CM; (**e**) HPMC; (**f**) CUR-MAG-HPMC CM; (**g**) HPC; (**h**) CUR-MAG-HPC CM; (**i**) PVP K30; (**j**) CUR-MAG-PVP K30 CM.

In order to further investigate and better understand molecular interactions of the binary and ternary co-amorphous systems, ss ^13^C NMR spectroscopy was conducted additionally. The ss ^13^C NMR spectra of crystalline CUR, crystalline MAG, CUR-MAG PM, CUR-MAG CM, CUR-MAG-HPMC CM, CUR-MAG-HPC CM, and CUR-MAG-PVP K30 CM are shown in Figure 6, and resonance assignments of CUR and MAG in ss ^13^C NMR spectra are presented in Table S3 with the assistance of ACD/Labs NMR Predictors software (Advanced Chemistry Development, Inc., Toronto, ON, Canada). For crystalline CUR, the resonances detected at 185.68 and 181.23 ppm were assigned to the signals of C12 and C15 (Figure 1a) in the C=O group. The resonances at 147.07 ppm corresponded to the signals of C1, C2, C21, and C22, which were bonded to the phenolic OH and OCH_3_ groups on the benzene ring (Figure 6a). In the ^13^C NMR spectrum of crystalline MAG, the resonance at 148.91 ppm was assigned to the signals of C1 and C9 (i.e., C1-OH and C9-OH) from the benzene ring (Figure 1b). The carbon signals of CH_2_ (i.e., C16 NS C20) were observed at 114.11 ppm (Figure 6b). The CUR-MAG PM exhibited a spectrum with overlapping carbon signals of crystalline CUR and MAG (Figure 6c).

However, CUR-MAG CM showed some shifted or disappeared signals with significantly broadening lines, which was distinguishable from the physical mixture (Figure 6d). On the one hand, the carbon signals (i.e., C12 and C15) from the C=O group disappeared and the carbon signals of C2 and C22 (i.e., C2-OH and C22-OH) shifted from 147.07 to 146.61 ppm in CUR. On the other hand, the carbon signals (i.e., C16 and C20) from the CH_2_ group shifted from 114.11 to 114.62 ppm in MAG. According to the results of spectral analysis (Raman, FTIR, and ^13^C NMR spectra), the intermolecular hydrogen bond interactions in CUR-MAG CM might occur between the phenolic OH and/or C=O groups in CUR and the CH_2_ group in MAG. In addition, the ternary co-amorphous systems exhibited similar spectra with CUR-MAG CM except for some new and weak signals (Figure 6f,h,j). In the ternary co-amorphous systems, the original signals of the binary CUR-MAG CM system did not change, indicating that the added polymers (HPMC, HPC, and PVP K30) could not break the molecular interaction between CUR and MAG in CUR-MAG CM. The generation of new signals in the ss ^13^C NMR spectra was due to the polymer introduction. As compared with the signals of polymers (Figure 6e,g,i), the new signals from polymers in ternary co-amorphous systems shifted to varying degrees. For example, the signals of PVP K30 at 171.93, 39.78, 28.15, and 15.16 ppm (Figure 6i) shifted to 176.66, 41.59, 30.92, and 16.32 ppm, respectively (Figure 6j). These results indicated that the added polymers involved molecular interactions in the ternary co-amorphous systems, but such interactions did not disturb the expected hydrogen bond formation between CUR and MAG.

### 3.4. Dissolution under Supersaturated Conditions

A co-amorphous system is a common approach to produce supersaturation in order to enhance gastrointestinal absorption. In a supersaturated system, higher drug concentration (above its equilibrium solubility) allows more free drugs in the solution state to achieve effective absorption in vivo [1,46]. Therefore, in vitro dissolution tests under supersaturated conditions were conducted in the simulated gastric fluid (0.1 M HCl buffer, pH 1.2) and simulated intestinal fluid (0.2 M phosphate buffer, pH 6.8), to observe the extent and duration of supersaturated dissolutions for CUR-MAG CM and its ternary systems.

**Figure 7:**
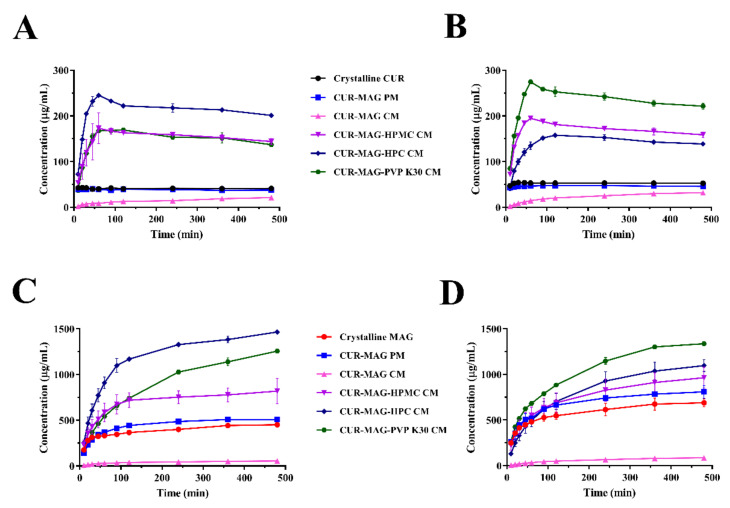
In vitro concentration–time profiles of CUR determined in (**A**) HCl buffer (pH = 1.2) and (**B**) phosphate buffer (pH = 6.8). In vitro concentration–time profiles of MAG determined in (**C**) HCl buffer (pH = 1.2) and (**D**) phosphate buffer (pH = 6.8).

As shown in Figure 7, crystalline CUR and CUR-MAG PM exhibited similar dissolution performance with a low concentration of CUR around 40–50 μg/mL (Figure 7A,B), and CUR-MAG PM performed slightly high MAG dissolution as compared with crystalline MAG in the two media (Figure 7C,D). However, unlike high dissolution of co-amorphous formulations widely reported, CUR-MAG CM exhibited much lower dissolution than their respective crystalline counterparts. The dissolution concentration of CUR in CUR-MAG CM only reached 21.17 μg/mL in pH 1.2 HCl buffer and 31.98 μg/mL in pH 6.8 phosphate buffer at 480 min. In addition, the maximum MAG concentration in CUR-MAG CM only achieved 54.19 μg/mL (pH 1.2) and 86.14 μg/mL (pH 6.8), respectively, which was much lower than the dissolution concentration of crystalline MAG during the whole dissolution process. The corresponding kinetic parameters including maximum concentration of dissolved drug (C_max_) and area under the dissolution curve (AUDC) are summarized in Table S4. The AUDC of CUR determined from CUR-MAG CM were only 36% and 45% that of crystalline CUR in pH 1.2 HCl buffer and pH 6.8 phosphate buffer within 480 min, respectively. As compared with crystalline MAG, the AUDC of MAG determined from CUR-MAG CM merely reached 11% and 10% that of its crystalline counterpart in two dissolution media, respectively.

For the modified ternary co-amorphous systems including CUR-MAG-HPMC CM, CUR-MAG-HPC CM, and CUR-MAG-PVP K30 CM, the higher C_max_ and AUDC were observed than those of crystalline CUR/MAG and CUR-MAG CM, indicating significant improvement in dissolution of both components (Figure 7 and Table S4). As compared with crystalline CUR, the CUR-MAG-HPMC CM, CUR-MAG-HPC CM, and CUR-MAG-PVP K30 CM showed significant enhancements (*p* < 0.01) in C_max_ (4.00-, 5.68- and 3.92-fold, respectively) and AUDC (3.72-, 5.21-, and 3.68-fold, respectively) in pH 1.2 HCl buffer, as well as in C_max_ (3.60-, 2.92-, and 5.08-fold, respectively) and AUDC (3.22-, 2.70-, 4.45-fold, respectively) in pH 6.8 phosphate buffer. In addition, as compared with crystalline MAG, the AUDC was increased by 1.82-, 3.12-, and 2.36-fold in pH 1.2 HCl buffer and 1.31-, 1.42-, and 1.79-fold in pH 6.8 phosphate buffer for CUR-MAG-HPMC CM, CUR-MAG-HPC CM, and CUR-MAG-PVP K30 CM, respectively.

Furthermore, the ternary co-amorphous systems showed a faster initial drug dissolution than CUR-MAG CM, and then achieved supersaturated concentration. In detail, the dissolved CUR concentration of CUR-MAG-HPMC CM, CUR-MAG-HPC CM, and CUR-MAG-PVP K30 CM increased gradually, reaching the C_max_ at 60, 60, and 120 min in pH 1.2 HCl buffer, as well as at 60, 120, and 60 min in pH 6.8 phosphate buffer, respectively (Figure 7A,B). After reaching the C_max_, the CUR concentration dropped slightly, but still maintained supersaturation for a long time. In addition, the ternary co-amorphous systems exhibited continuous supersaturated dissolution of MAG without decreasing concentration (Figure 7C,D). Whereas, for CUR-MAG-MCC CM, CUR-MAG-VA64 CM, and CUR-MAG-S630 CM, they showed similar or slightly higher dissolution behavior, with no significant improvement in the dissolution of CUR-MAG CM (Figure S6).

### 3.5. Contact Angle Measurements and Surface Free Energy Calculation

Visually, CUR-MAG CM aggregated into clumps and sank to the bottom during dissolution (Figure S7A), the CUR-MAG CM co-formulated with MCC, VA64, or S630 showed similar aggregation behavior (Figure S7E–G), while the ternary co-amorphous systems containing HPMC, HPC, or PVP K30 did not aggregate and easily dispersed or suspended in the medium (Figure S7B–D). After dissolution of CUR-MAG CM, CUR-MAG-MCC CM, CUR-MAG-VA64 CM, and CUR-MAG-S630 CM, the residual solid appeared hard when touched by hand (Figure S8). Combined with the dissolution performance, it was speculated that aggregation of CUR-MAG CM during the dissolution process was the main reason for the significantly decreased dissolution. According to the Noyes–Whitney dissolution model, the serious aggregation reduced the surface area, and thus limited drug dissolution. The addition of HPMC, HPC, and PVP K30 into CUR-MAG CM eliminated such aggregation, thus, improving its dissolution.

**Figure 8:**
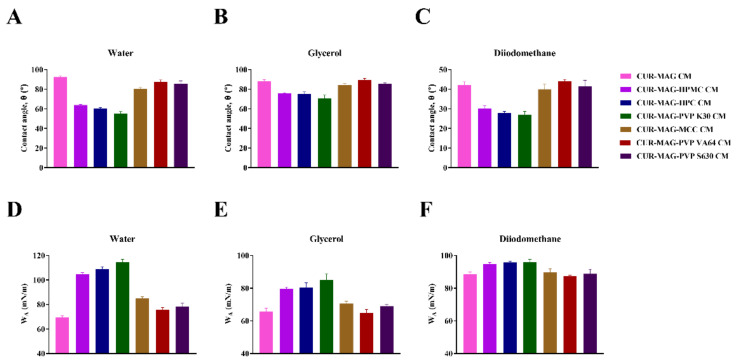
The contact angle (*θ*) of co-amorphous samples using (**A**) water, (**B**) glycerol and (**C**) diiodomethane. The work of the liquid adhesion to solid surface (W_A_) of co-amorphous samples using (**D**) water, (**E**) glycerol and (**F**) diiodomethane.

Contact angle is a common technique to investigate the wettability of powders. As seen from Figure 8, the water contact angle of CUR-MAG CM was 92.5° (*θ* > 90°) (Figure 8A), indicating that CUR-MAG CM was hydrophobic [52,53]. While, CUR-MAG-HPMC CM, CUR-MAG-HPC CM, and CUR-MAG-PVP K30 CM exhibited water contact angles with 63.9°, 60.3° and 55.1° (*θ* < 90°), respectively, suggesting the polymer addition changed the surface properties of CUR-MAG CM from hydrophobicity to hydrophilicity. In contrast, CUR-MAG CM co-formulated with MCC, VA64 and S630 showed similar contact angles to CUR-MAG CM. For other test liquids (glycerol and diiodomethane), the contact angles showed a similar trend with the water test for co-amorphous samples (Figure 8B,C).

The adhesion work of a liquid to a solid surface (W_A_) is determined from Equation (9). Wetting may occur when a liquid remains in contact with a solid surface. The degree of wetting is determined by the cohesion between the liquid molecules and the adhesion forces resulting from the molecular interactions between the liquid and the solid [54,55]. The greater the W_A_, the more the liquid can wet the solid [36]. The calculated value of W_A_ (water to the surface of CUR-MAG CM) was 69.7 mN/m. For CUR-MAG-MCC CM, CUR-MAG-VA64 CM, and CUR-MAG-S630 CM, the adhesion work of water to their surface exhibited a similar or slight improvement to CUR-MAG CM, while, for CUR-MAG-HPMC CM, CUR-MAG-HPC CM, and CUR-MAG-PVP K30 CM, their W_A_ values were 104.9, 108.9, and 114.5 mN/m, respectively. The significantly increased W_A_ values indicated that co-formulating with HPMC, HPC, or PVP K30 could effectively improve the wetting ability of CUR-MAG CM (Figure 8D).

**Figure 9:**
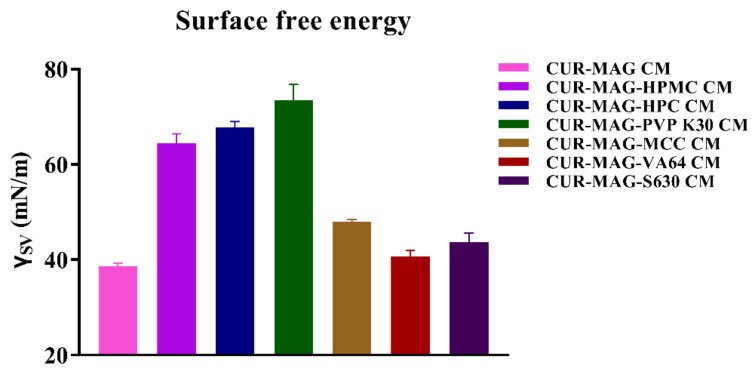
Surface free energy of co-amorphous samples calculated according to van Oss method.

Furthermore, the surface free energy was calculated from the contact angle data by van Oss’s acid-base method [38,39]. Taking into account the obtained values of the contact angle of standard test liquids (Table S5) [38,56], the surface free energy of CUR-MAG CM was 38.6 mN/m and the polymer addition (MCC, VA64, and S630) showed a similar or slightly increased surface free energy. In contrast, CUR-MAG-HPMC CM, CUR-MAG-HPC CM, and CUR-MAG-PVP K30 CM exhibited higher surface free energy (64.4, 67.7, and 73.5 mN/m, respectively) (Figure 9). For the wetting ability of a solid, the higher the solid surface energy or the smaller the liquid surface tension, the greater the wetting ability of a solid [54]. Thus, wettability of a solid surface can be increased either by lowering the surface tension of the liquid or by increasing the surface free energy of the solid [34,38]. In this study, a small amount of polymer addition (HPMC, HPC, and PVP K30) increased the surface free energy of CUR-MAG CM, and hence enhanced its wettability. The improvement of surface free energy and wettability promoted the infiltration of water into the powder of CUR-MAG CM and facilitated the dispersion of the drug powder (i.e., deaggregating CUR-MAG CM) during dissolution, so as to effectively enhance its dissolution.

### 3.6. Nucleation Inhibitory Effect on CUR and MAG by Polymer

Given the thermodynamic force driven for nuclear formation and growth, when the drug concentration is higher than its equilibrium solubility, supersaturation would lead to the reprecipitation/recrystallization of dissolved drugs in the dissolution media [19,46]. Due to the important effect of supersaturation on nucleation kinetics, it is appropriate to compare the nucleation time under the same supersaturation condition. Thus, nucleation inhibitory effect on CUR and MAG by polymer was measured at the same concentration of CUR for the determination of nucleation induction time (t_n_).

**Figure 10:**
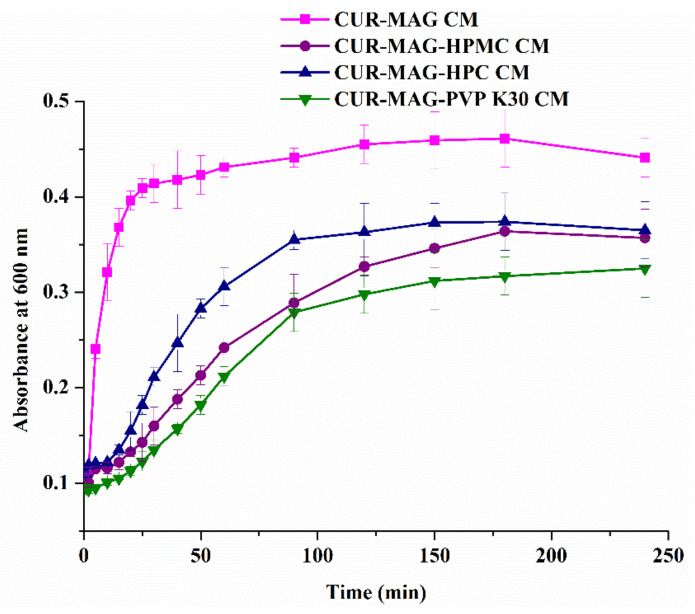
Nucleation inhibitory effect on CUR and MAG by polymer in water.

The turbidity-time profile of drugs after adding the prepared DMSO solution into water was determined using a UV spectrophotometer. The solution was clear initially, and a baseline or minimum turbidity was recorded. Gradually, the solution became turbid arising from the formed nuclei and developed into macroscopic crystals over time. According to the results, the profile of solution turbidity versus time was plotted, and t_n_ was determined by drawing regression lines through the two distinct linear regions and taking their intersection point as its value [57,58]. When the DMSO solution of CUR-MAG CM was added into water, the turbidity increased rapidly in the first 30 min and reached the maximum absorbance at about 180 min (Figure 10). The calculated t_n_ of CUR-MAG CM in water was 1.94 ± 0.85 min. However, when adding DMSO solution dissolving CUR-MAG-HPMC CM, CUR-MAG-HPC CM, and CUR-MAG-PVP K30 CM at the same CUR concentration, the aqueous solutions remained clear at first, and then slowly became cloudy, and the turbidity profiles gradually changed from steep to gentle. The t_n_s were prolonged to 21.23 ± 5.33 min, 14.31 ± 3.12 min, and 22.05 ± 3.91 min (10.9-, 7.4-, and 11.4-fold of CUR-MAG CM), respectively (*n* = 3), indicating that polymer addition could inhibit the nucleation of CUR and MAG in water. In general, polymers acted as crystallization inhibitors in the dissolution medium, effectively maintaining the supersaturation of drugs [41,59,60]. In addition, the precipitated product was obtained by centrifugation after the nucleation study of CUR-MAG CM, and then vacuum-dried for 24 h. The XRPD pattern of the precipitated product was compared to those of crystalline CUR/MAG (Figure S9). The diffraction peaks of the precipitated product were mainly from crystalline CUR. Therefore, the crystallized CUR from the supersaturated solution of CUR-MAG CM was form I of CUR.

The proposed mechanism of deaggregation and crystallization inhibition by polymer addition in enhancing the dissolution of CUR-MAG CM is schematically depicted in Figure S10. When contacting the dissolution medium, the surface of CUR-MAG CM acted as a hydrophobic layer, and it was difficult for water to enter or disperse, resulting in the agglomeration of CUR-MAG CM and encapsulation of the powder which was not in contact with water, thus, causing the much lower dissolution of CUR-MAG CM. After forming ternary co-amorphous system, the polymer addition (5% of HPMC, HPC, or PVP K30) changed the group distribution (an increase in its polar component) on the surface of CUR-MAG CM (from hydrophobic to hydrophilic) [61], increased the wetting ability and surface free energy of CUR-MAG CM, and hence deaggregated CUR-MAG CM during dissolution, resulting in the significantly improved dissolution. If the added polymer had no significant improvement in the wettability and surface free energy of CUR-MAG CM, the polymer (such as MCC, VA64, and S630) would not produce the deaggregating effect and not enhance its dissolution performance.

### 3.7. BDS

**Figure 11:**
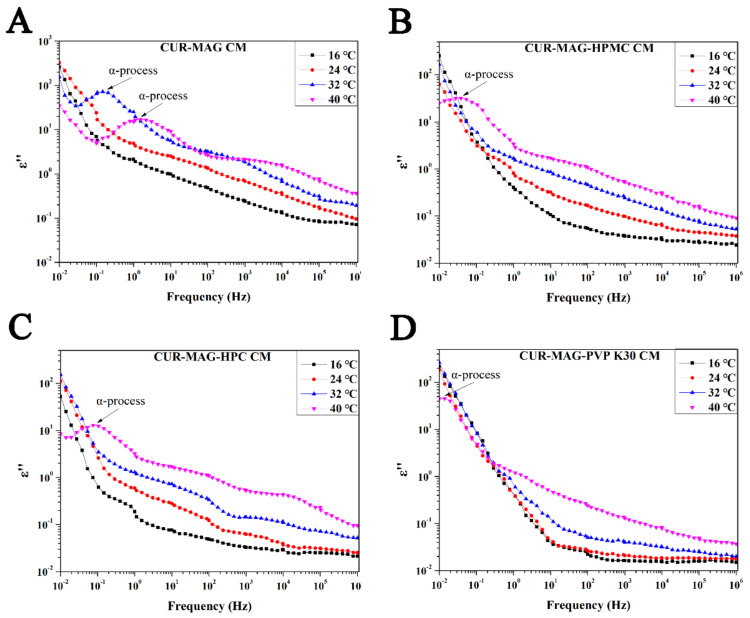
Dielectric loss spectra:(**A**) CUR-MAG CM; (**B**) CUR-MAG-HPMC CM; (**C**) CUR-MAG-HPC CM; (**D**) CUR-MAG-PVP K30 CM.

The origin of structural relaxation is considered to be the synergistic movement between molecules, and α-relaxation plays a leading role in structural relaxation and molecular mobility because it involves a wide range of particle movements [62,63]. The dielectric spectroscopy was performed to investigate the molecular mobility of CUR-MAG CM in the presence and absence of very little polymer additives. As shown in Figure 11, dielectric loss spectra of CUR-MAG CM, CUR-MAG-HPMC CM, CUR-MAG-HPC CM, and CUR-MAG-PVP K30 CM from 16 to 40 °C (containing the respective T_g_) revealed well-resolved peaks of α-relaxation, which was a measure of the global molecular mobility. For CUR-MAG CM, no α-relaxation was detected at 16 and 24 °C, and the α-relaxation peaks shifted to higher frequencies with increased temperature from 32 to 40 °C (Figure 11A), indicating faster global molecular motions. In the ternary co-amorphous systems, no α-relaxation occurred at 16, 24, and 32 °C and α-relaxation only occurred at 40 °C in the detected frequency range (Figure 11B–D). As compared with CUR-MAG CM, the α-relaxation process of the ternary co-amorphous systems shifted from high frequency to low frequency at 40 °C in the order of CUR-MAG CM, CUR-MAG-HPC CM, CUR-MAG-HPMC CM, and CUR-MAG-PVP K30 CM, indicating that a small amount of polymer addition significantly inhibited the molecular mobility of CUR-MAG CM [63].

### 3.8. Physical Stability Evaluation

**Figure 12:**
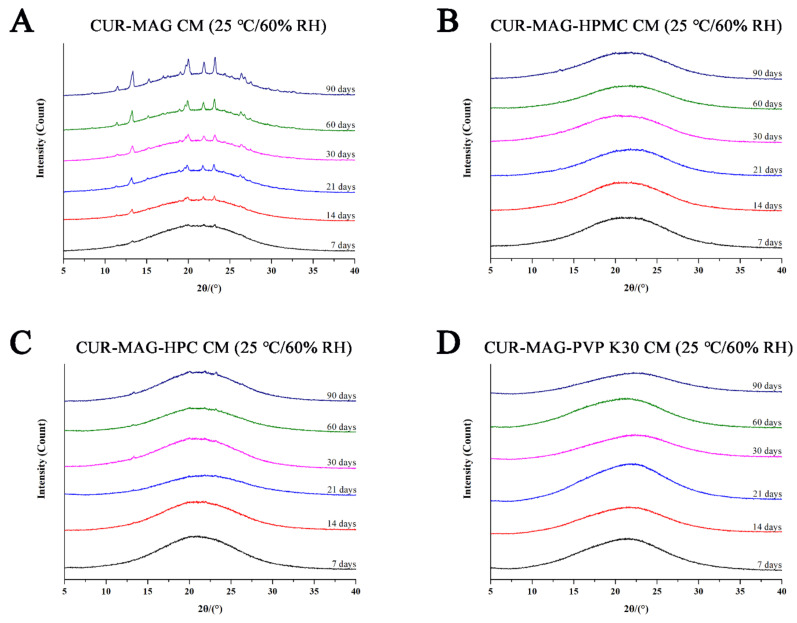
XRPD diffractograms: (**A**) CUR-MAG CM; (**B**) CUR-MAG-HPMC CM; (**C**) CUR-MAG-HPC CM; (**D**) CUR-MAG-PVP K30 CM stored at 25 °C/60% RH condition for different times.

Physical stability is the crucial quality attribute of amorphous formulations. Thus, the physical stability of CUR-MAG CM and its ternary systems was evaluated at 25 °C/60% RH and 40 °C/75% RH using XRPD analysis. As seen from Figure 12, several diffraction peaks were observed in the XRPD pattern of CUR-MAG CM after 7 days at 25 °C/60% RH, and the peak intensity gradually increased within 90 days, indicating more recrystallization of CUR-MAG CM (Figure 12A). While almost no characteristic diffraction peaks were observed in the XRPD patterns of CUR-MAG-HPMC CM, CUR-MAG-HPC CM, and CUR-MAG- PVP K30 CM at 25 °C/60% RH throughout 90 days (Figure 12B–D), indicating the absence or slow rate of crystalline transformation, and hence the prominently enhanced stability.

**Figure 13:**
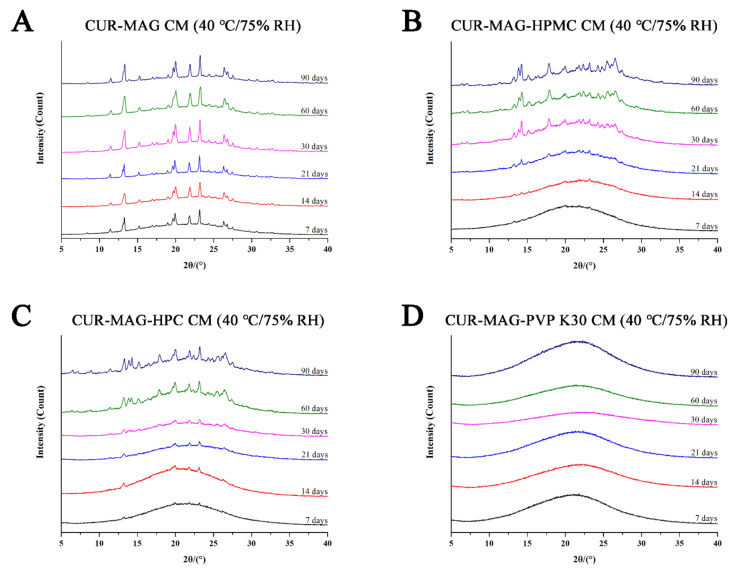
XRPD diffractograms: (**A**) CUR-MAG CM; (**B**) CUR-MAG-HPMC CM; (**C**) CUR-MAG-HPC CM; (**D**) CUR-MAG-PVP K30 CM stored at 40 °C/75% RH condition for different times.

Furthermore, the physical stabilities of CUR-MAG CM, CUR-MAG-HPMC CM, CUR-MAG-HPC CM, and CUR-MAG-PVP K30 CM were further evaluated under 40 °C/75% RH condition. As shown in Figure 13, the XRPD pattern of CUR-MAG CM showed some diffraction peaks at 7 days and the peak intensity got stronger with increasing days (Figure 13A). Meanwhile, CUR-MAG-HPMC CM and CUR-MAG-HPC CM also showed increasing peak intensity during the whole period, but the intensity was much lower than that of CUR-MAG CM (Figure 13B,C). In addition, the diffractogram of CUR-MAG-PVP K30 CM still retained an amorphous halo, indicating its superior physical stability as compared with CUR-MAG-HPMC CM and CUR-MAG-HPC CM (Figure 13D). Therefore, these results confirmed that the stability of CUR-MAG CM could be improved by adding a small amount of polymer. The added polymers could disperse well in CUR-MAG CM due to the good miscibility between them, and potentially interacted with CUR-MAG CM in the ternary systems and inhibited its molecular mobility, thus, effectively inhibiting the recrystallization of CUR-MAG CM [41,64,65].

In addition, several strong diffraction peaks were observed in the XRPD pattern of the residual solid, which was collected from CUR-MAG CM dissolved at 240 min, and the peak intensity increased at 480 min (Figure S11), indicating that recrystallization occurred in CUR-MAG CM during dissolution. However, no diffraction peaks or low intensity peaks were observed in the XRPD patterns of the collected samples from CUR-MAG-HPMC CM, CUR-MAG-HPC CM, and CUR-MAG-PVP K30 CM at 240 min and even 480 min, indicating higher physical stability of their undissolved samples. In the ternary co-amorphous systems, the added polymer served as a crystallization inhibitor for CUR-MAG CM and effectively inhibited nucleation and crystal growth, and hence slowed down the recrystallization of CUR-MAG CM [10,66].

## 4. Conclusions

**Figure 14:**
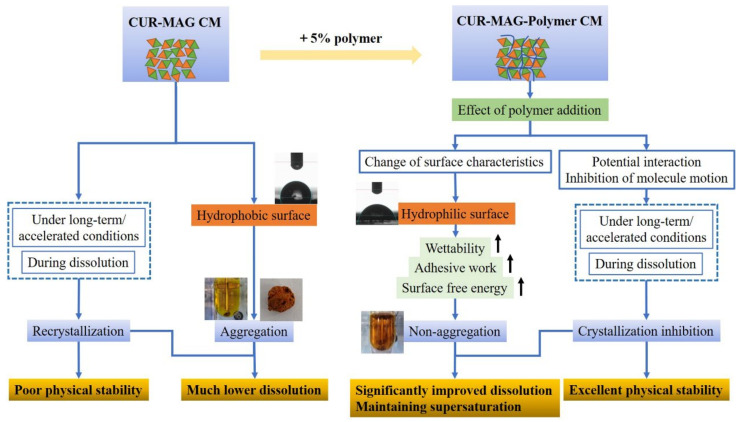
Summary of deaggregation and crystallization inhibition by polymer addition in optimizing the dissolution and physical stability of CUR-MAG CM.

In the present study, polymer addition improved the wetting ability of CUR-MAG CM, enhanced its surface free energy, and hence eliminated the aggregation of CUR-MAG CM during dissolution, resulting in significantly improved dissolution. Moreover, the ternary co-amorphous systems showed superior physical stabilities as compared with CUR-MAG CM, attributed to the involved molecular interactions and molecular motion inhibition by polymer addition (Figure 14). Such ternary co-amorphous systems avoid some of the limitations associated with polymer-based ASD, such as a limited drug load, poor physical stability caused by potential immiscibility between a drug and polymer, and/or the hygroscopicity of many polymers. In summary, this study offers a promising approach to optimize co-amorphous systems with low quality and performance for developing robust co-amorphous drug products.

## Figures and Tables

**Table 1:** The experimental and theoretical *T_g_* values of CUR-MAG CM and its ternary co-amorphous systems.

Sample	Experimental *T_g_*, °C	Calculated *T_g_*, °C	*ΔT_g_*, °C
Amorphous CUR	74.6		
Amorphous MAG	−16.4		
CUR-MAG CM	21.1	29.5	−8.4
CUR-MAG-HPMC CM	26.1	22.0	4.1
CUR-MAG-HPC CM	23.5	21.9	1.6
CUR-MAG-PVP K30 CM	37.4	22.0	15.4

## Data Availability

Not applicable.

## Supplementary Materials

The following are available online at https://www.mdpi.com/article/10.3390/pharmaceutics13101725/s1, Figure S1: TGA charts of crystalline CUR (A) and crystalline MAG (B), Figure S2: PLM photographs of (a) crystalline CUR, (b) crystalline MAG, (c) CUR-MAG CM, (d) CUR-MAG-HPMC CM, (e) CUR-MAG-HPC CM, (f) CUR-MAG-PVP K30 CM, (g) CUR-MAG-MCC CM, (h) CUR-MAG-VA64 CM and (i) CUR-MAG-S630 CM, Figure S3: Comparison of the experimental pattern of crystalline CUR with the standard pattern from the Cambridge Crystallographic Data Centre, Figure S4: Comparison of the experimental pattern of crystalline MAG with the standard pattern from the Cambridge Crystallographic Data Centre, Figure S5: DSC thermograms of amorphous CUR and amorphous MAG prepared in situ using differential scanning calorimetry, Figure S6: In vitro concentration–time profiles of CUR determined in (A) HCl buffer (pH = 1.2) and (B) phosphate buffer (pH = 6.8), and in vitro concentration–time profiles of MAG determined in (C) HCl buffer (pH = 1.2) and (D) phosphate buffer (pH = 6.8) for the dissolution of CUR-MAG-MCC CM, CUR-MAG-VA64 and CUR-MAG-S630 CM, Figure S7: Photographs of (A) CUR-MAG CM, (B) CUR-MAG-HPMC CM, (C) CUR-MAG-HPC CM, (D) CUR-MAG-PVP K30 CM, (E) CUR-MAG-MCC CM, (F) CUR-MAG-VA64 CM and (G) CUR-MAG-S630 CM during dissolution at 240 min in pH 6.8 phosphate buffer, Figure S8: Photographs of (A) CUR-MAG CM, (B) CUR-MAG-MCC CM, (C) CUR-MAG-VA64 CM and (D) CUR-MAG-S630 CM after dissolution in pH 6.8 phosphate buffer, Figure S9: Comparison of the XRPD patterns between the precipitated product and crystalline CUR/MAG. Figure S10: Proposed mechanism of deaggregation and crystallization inhibition by polymer addition in improving the dissolution of CUR-MAG CM, Figure S11: XRPD diffractograms of CUR-MAG CM, CUR-MAG-HPMC CM, CUR-MAG-HPC CM, and CUR-MAG-PVP CM during dissolution in pH 6.8 phosphate buffer at (A) 240 min and (B) 480 min; Table S1: Theoretical calculation of solubility parameters; Table S2: The yields of CUR-MAG CM and its ternary co-amorphous systems; Table S3: Resonance assignments of CUR and MAG in ^13^C NMR spectra in solid state; Table S4: Kinetic parameters including maximum concentration of dissolved drug (C_max_) and area under dissolution curve (AUDC) determined from the drug dissolution profiles; Table S5: Values of the Lifshitz-van der Waals ($\gamma^{LW}$) and acid-base ($\gamma^{AB}$) components and electron-acceptor ($\gamma^{+}$) and electron-donor ($\gamma^{-}$) parameters of the acid-base component of the surface tension ($\gamma^{tot}$) of the model liquids, used for the surface free energy determination of co-amorphous samples.
