# Supplementary material for: Deaggregation and Crystallization Inhibition by Small Amount of Polymer Addition for a Co-Amorphous Curcumin-Magnolol System
Source: Pharmaceutics. 2021 Oct 18;13(10):1725. doi: 10.3390/pharmaceutics13101725 (PMC8540313; doi:10.3390/pharmaceutics13101725)
Supplement: Supplementary file 1 [file pharmaceutics-13-01725-s001.zip › pharmaceutics-1367511-supplementary.pdf]

pharmaceutics

Supplementary Materials: Deaggregation and Crystallization Inhibition by Small Amount of Polymer Addition for a Co-Amorphous Curcumin-Magnolol System

Jiawei Han, Luyuan Li, Meiling Su, Weili Heng, Yuanfeng Wei, Yuan Gao and Shuai Qian

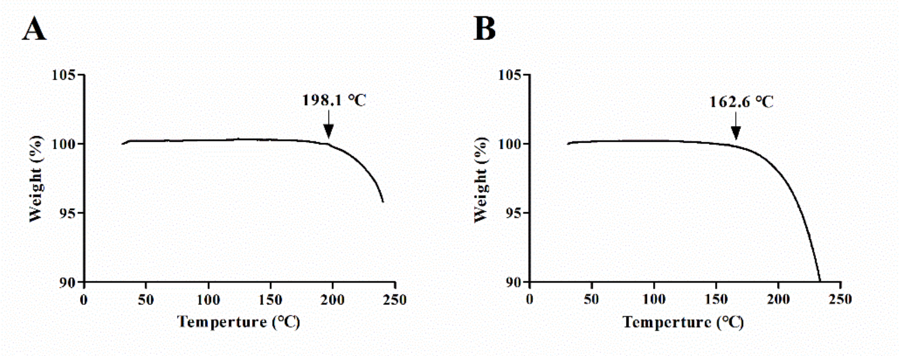

Figure S1. TGA charts of crystalline CUR (A) and crystalline MAG (B).

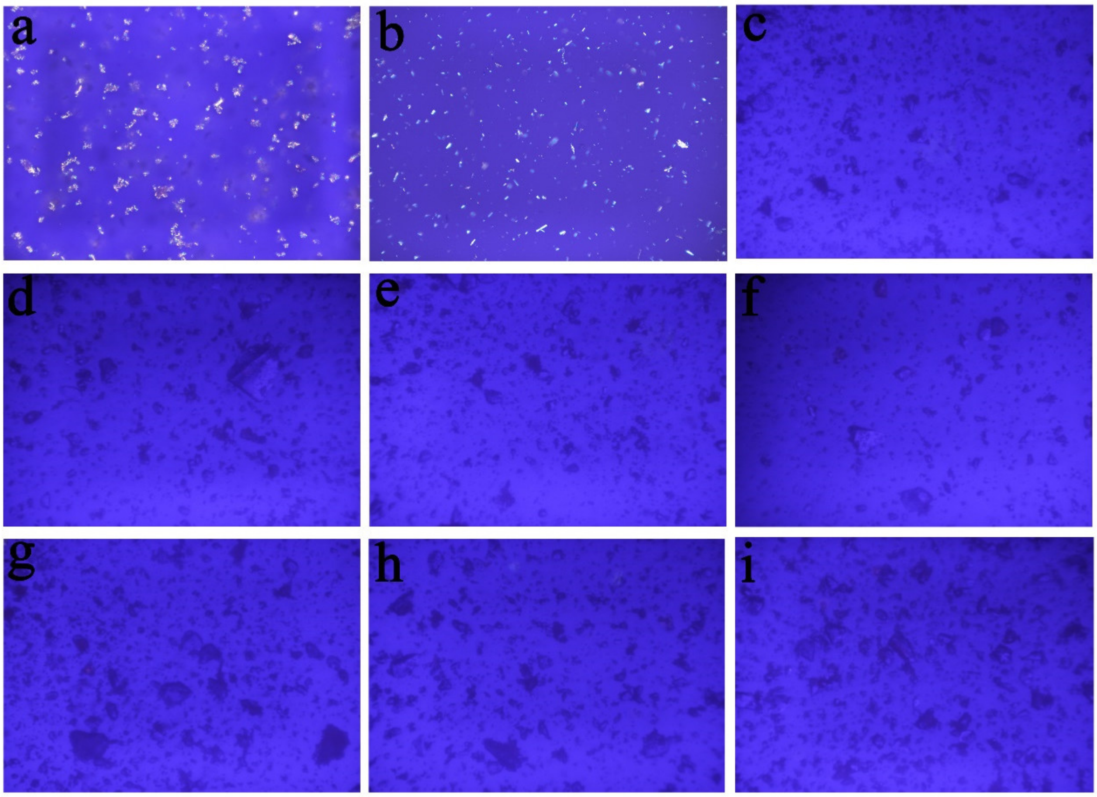

Figure S2. PLM photographs of (a) crystalline CUR, (b) crystalline MAG, (c) CUR-MAG CM, (d) CUR-MAG-HPMC CM, (e) CUR-MAG-HPC CM, (f) CUR-MAG-PVP K30 CM, (g) CUR-MAG-MCC CM, (h) CUR-MAG-VA64 CM and (i) CUR-MAG-S630 CM.

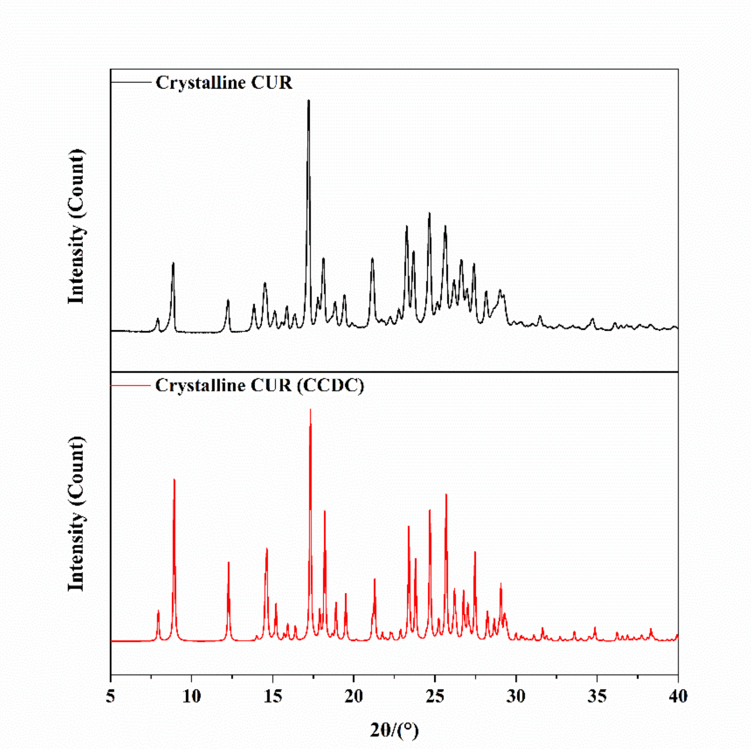

Figure S3. Comparison of the experimental pattern of crystalline CUR with the standard pattern from the Cambridge Crystallographic Data Centre.

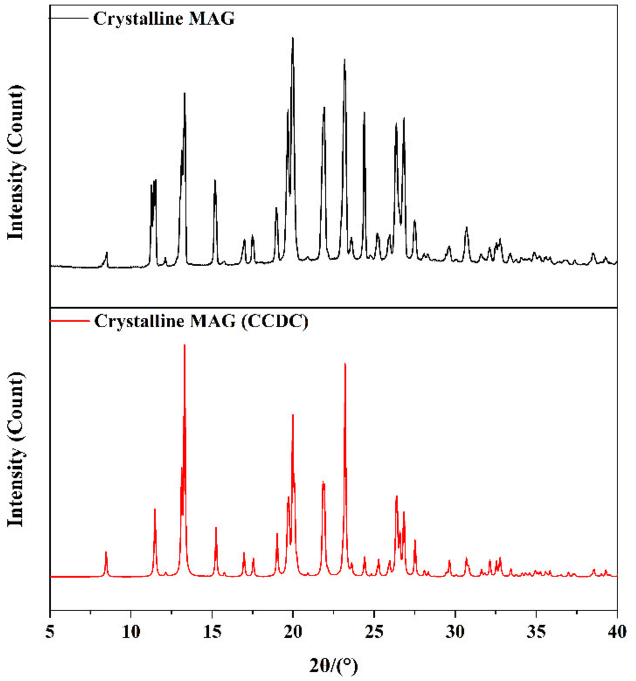

Figure S4. Comparison of the experimental pattern of crystalline MAG with the standard pattern from the Cambridge Crystallographic Data Centre.

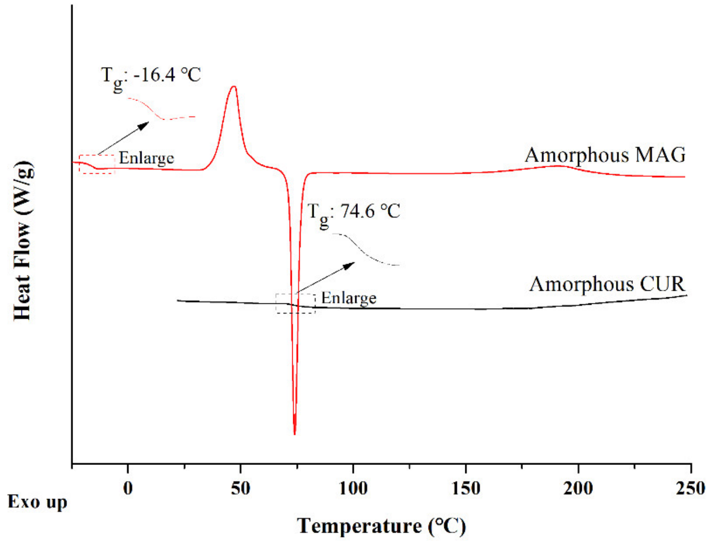

Figure S5. DSC thermograms of amorphous CUR and amorphous MAG prepared *in situ* using differential scanning calorimetry.

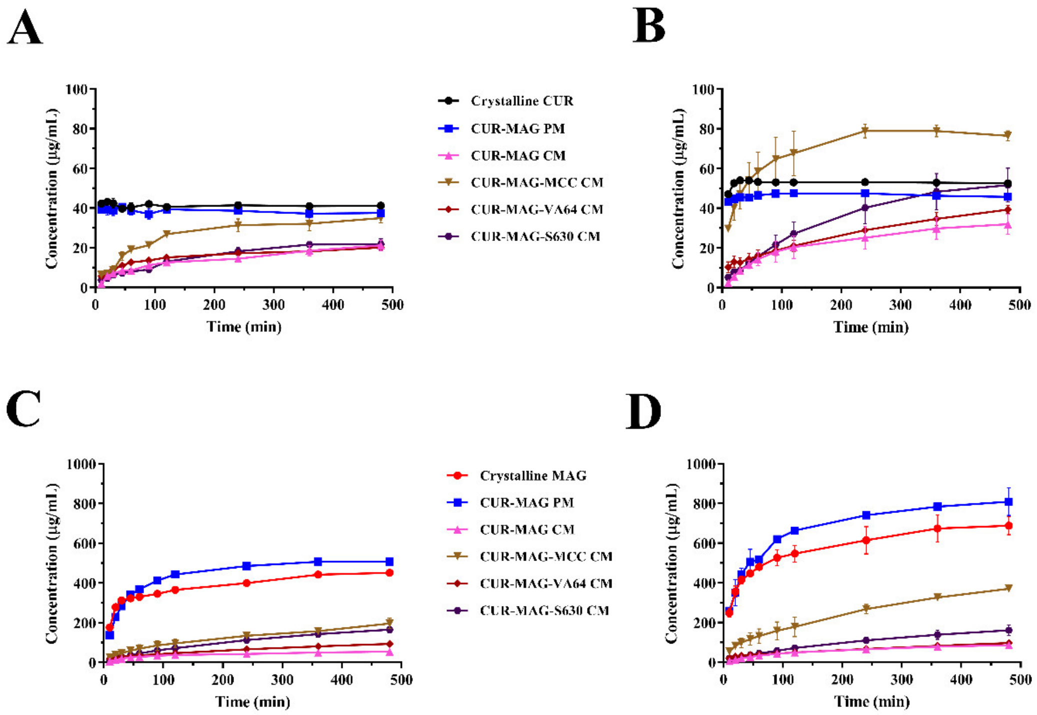

Figure S6. *In vitro* concentration - time profiles of CUR determined in (A) HCl buffer (pH = 1.2) and (B) phosphate buffer (pH = 6.8), and *in vitro* concentration - time profiles of MAG determined in (C) HCl buffer (pH = 1.2) and (D) phosphate buffer (pH = 6.8) for the dissolution of CUR-MAG-MCC CM, CUR-MAG-VA64 and CUR-MAG-S630 CM.

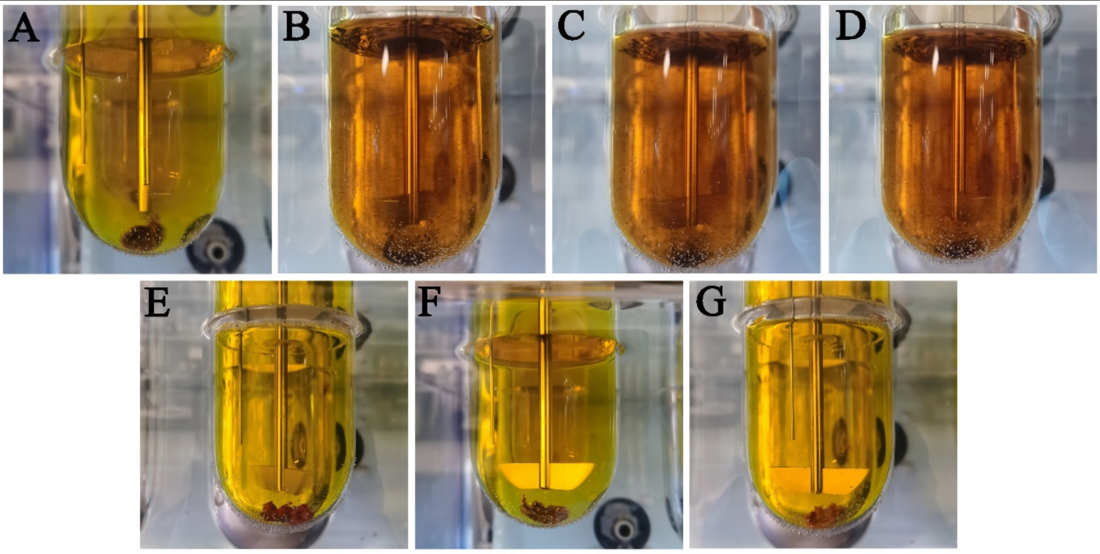

Figure S7. Photographs of (A) CUR-MAG CM, (B) CUR-MAG-HPMC CM, (C) CUR-MAG-HPC CM, (D) CUR-MAG-PVP K30 CM, (E) CUR-MAG-MCC CM, (F) CUR-MAG-VA64 CM and (G) CUR-MAG-S630 CM during dissolution at 240 min in pH 6.8 phosphate buffer.

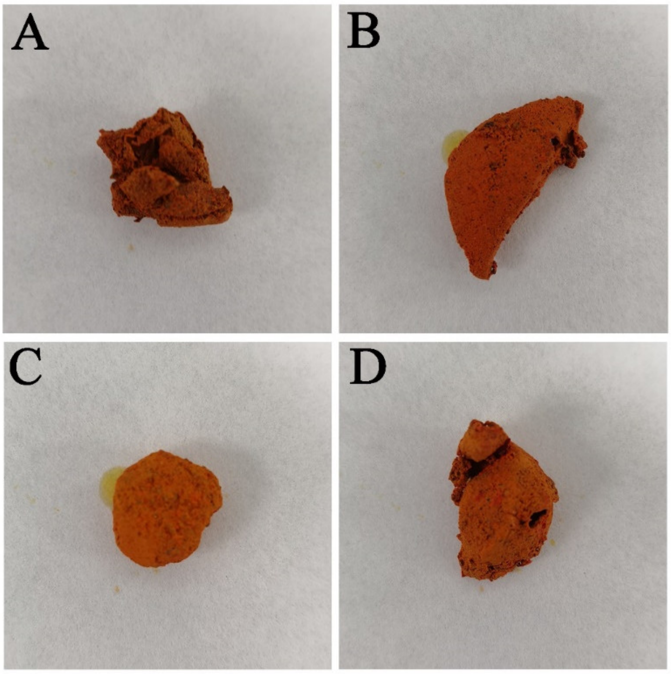

Figure S8. Photographs of (A) CUR-MAG CM, (B) CUR-MAG-MCC CM, (C) CUR-MAG-VA64 CM and (D) CUR-MAG-S630 CM after dissolution in pH 6.8 phosphate buffer.

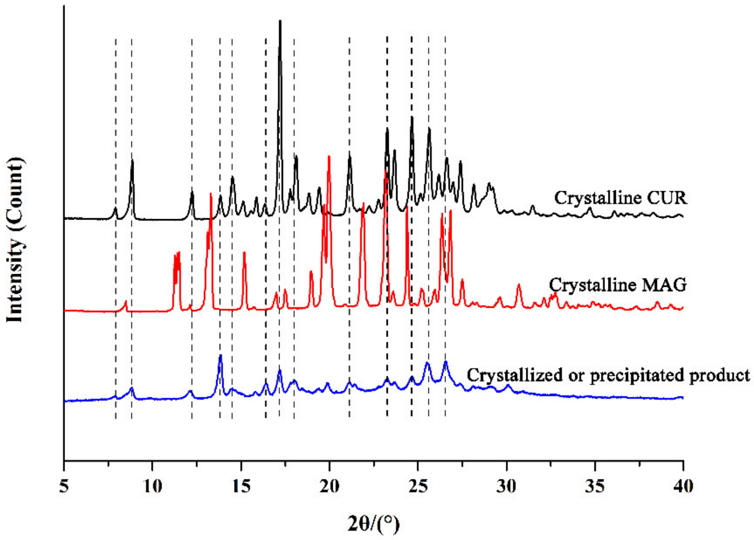

Figure S9. Comparison of the XRPD patterns between the precipitated product and crystalline CUR/MAG.

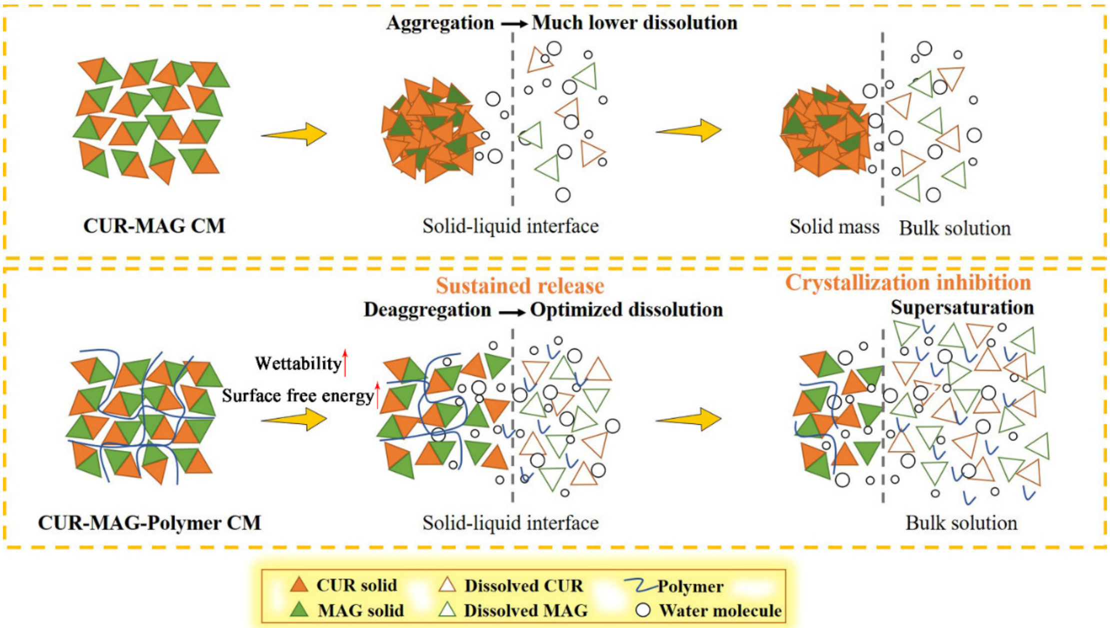

Figure S10. Proposed mechanism of deaggregation and crystallization inhibition by polymer addition in improving the dissolution of CUR-MAG CM.

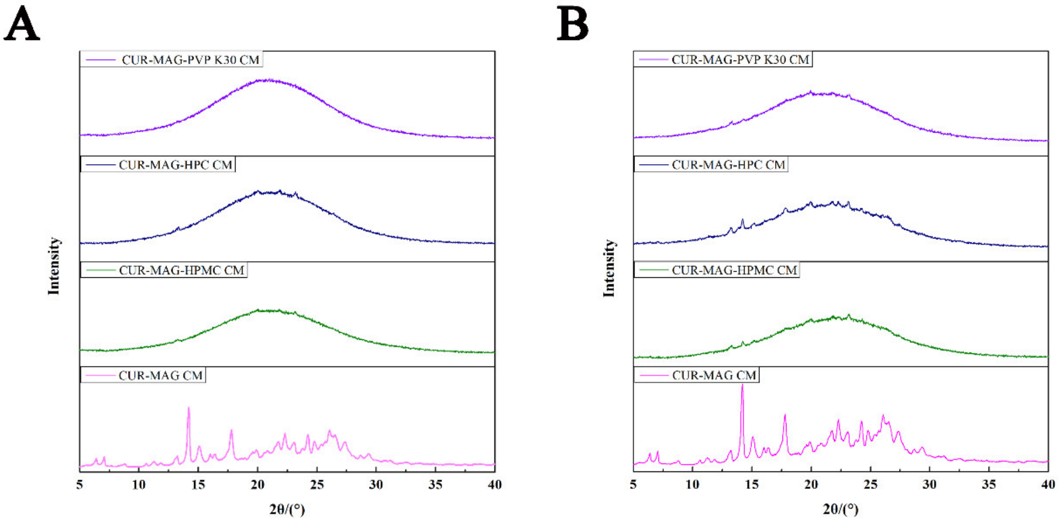

Figure S11. XRPD diffractograms of CUR-MAG CM, CUR-MAG-HPMC CM, CUR-MAG-HPC CM and CUR-MAG-PVP CM during dissolution in pH 6.8 phosphate buffer at (A) 240 min and (B) 480 min.

Table S1. Theoretical calculation of solubility parameters.

Components	δ ($MPa^{1/2}$)			Δδ ($MPa^{1/2}$)	
	Fedors method	Hoftyzer-Van Krevelen method	Mean δ ($MPa^{1/2}$)		
CUR	22.82	26.33	24.58		0.41
MAG	21.09	27.24	24.17	0.41	
HPMC	21.05	24.63	22.84	1.74	1.33
HPC	25.84	28.22	27.03	2.45	2.86
PVP K30	23.81	21.81	22.81	1.77	1.36
MCC	25.96	29.92	27.94	3.36	3.77
VA64	18.44	19.58	19.01	5.57	5.16
S630	19.04	20.79	19.92	4.66	4.25

Table S2. The yields of CUR-MAG CM and its ternary co-amorphous systems.

Samples	CUR-MAG CM	CUR-MAG-HPMC CM	CUR-MAG-HPC CM	CUR-MAG-PVP K30 CM
Yield	98.15 ± 0.74%	97.84 ± 1.43%	97.36 ± 1.92%	98.01 ± 0.80%

Table S3. Resonance assignments of CUR and MAG in ^{13}C NMR spectra in solid state.

Compound	Carbon Number	Chemical shift (ppm)
CUR	12, 15	185.69, 181.23
	1, 2, 21, 22	147.07
	10, 18	138.83
	11, 17	128.11
	5, 19	123.92
	4, 24	121.43
	3, 23	113.56
	6, 20	108.50
	8, 26	55.98
	14	54.00
MAG	1, 9	148.91
	15, 19	136.72
	4, 12	134.18
	3, 13	130.87
	5, 11	128.20
	2, 8	124.47
	6, 10, 16, 20	114.11
	14, 18	39.73, 36.14

pharmaceutics

MDPI

Table S4. Kinetic parameters including maximum concentration of dissolved drug (C_{max}) and area under dissolution curve (AUDC) determined from the drug dissolution profiles.

Samples	Measured component	C_{max} (μg/mL)	AUDC (pH 1.2) (min·μg/mL)	Relative percentage of AUDC	C_{max} (μg/mL)	AUDC (pH 6.8) (min·μg/mL)	Relative percentage of AUDC
Crystalline CUR	CUR	43.15 ± 1.35	19346 ± 171	100%	54.04 ± 0.39	24889 ± 98	100%
CUR-MAG PM		40.59 ± 1.80	17977 ± 233	93%	47.45 ± 1.38	21926 ± 218*	88%
CUR-MAG CM		21.17 ± 2.58**	6988 ± 277**	36%	31.98 ± 5.08**	11260 ± 825**	45%
CUR-MAG-HPMC CM		172.78 ± 33.87**	71912 ± 1065**	372%	194.69 ± 4.18**	80089 ± 838**	322%
CUR-MAG-HPC CM		245.22 ± 3.41**	100798 ± 955**	521%	157.70 ± 1.48**	67229 ± 658**	270%
CUR-MAG-PVP K30 CM		169.01 ± 4.52**	71163 ± 1121**	368%	274.47 ± 3.30**	110655 ± 1174**	445%
Crystalline MAG	MAG	450.96 ± 7.65	185660 ± 1484	100%	688.23 ± 45.37	280221 ± 8968	100%
CUR-MAG PM		507.41 ± 14.02*	215090 ± 1833*	116%	808.41 ± 69.58*	329391 ± 4602*	118%
CUR-MAG CM		54.19 ± 3.91**	19715 ± 430**	11%	86.14 ± 9.86**	29104 ± 1321**	10%
CUR-MAG-HPMC CM		818.57 ± 138.62**	337443 ± 13384**	182%	963.61 ± 115.29**	366711 ± 17019*	131%
CUR-MAG-HPC CM		1463.76 ± 27.49**	578949 ± 4919**	312%	1095.84 ± 64.26**	397400 ± 13842**	142%
CUR-MAG-PVP K30 CM		1255.93 ± 26.05**	437614 ± 4670**	236%	1335.05 ± 25.79**	500254 ± 4330**	179%

Each data represents the mean ± the standard deviation ($n = 3$). $^{*}p < 0.05$, $^{**}p < 0.01$, compared to the crystalline drug.

Table S5. Values of the Lifshitz-van der Waals (γ^{LW}) and acid-base (γ^{AB}) components and electron-acceptor (γ^{+}) and electron-donor (γ^{-}) parameters of the acid-base component of the surface tension (γ^{tot}) of the model liquids, used for the surface free energy determination of co-amorphous samples.

Liquid	γ^{tot}	γ^{LW}	γ^{AB}	γ^{+}	γ^{-}
Water	72.8	27.0	45.8	22.9	22.9
Glycerol	64.0	34.0	30.0	3.92	57.4
Diiodomethane	50.8	50.80			
